# A qualitative study of the causes and circumstances of drowning in Uganda

**DOI:** 10.1186/s12889-022-14461-6

**Published:** 2022-11-05

**Authors:** Anthony Buyinza Mugeere, Frederick Oporia, Olive Kobusingye

**Affiliations:** 1 Department of Sociology & Anthropology, School of Social Sciences, Makerere University, P.O. Box 7062, Kampala, Uganda; 2 School of Public Health, Makerere University, Kampala, Uganda

**Keywords:** Drowning, Prevention, Burden, Qualitative, Uganda

## Abstract

**Background:**

Drowning is a serious worldwide and preventable injury problem, especially in low- and middle-income countries (LMICs). The aim of this paper is to draw on the results of semi-structured interviews with witnesses, family members and friends of persons involved in fatal and nonfatal drowning incidents to describe the circumstances of drowning in both lakeside and non-lakeside districts and to identify potential contextually appropriate interventions for drowning prevention and surveillance in Uganda.

**Methods:**

The findings presented in this study were based on data collected from study participants selected through purposive sampling comprising 324 individual face-to-face interviews with drowning witnesses, family members, friends of and survivors of drowning and ten (10) focus group discussions held with community members in 14 districts in Uganda. Data analysis was done using the Framework Analysis Approach with the aid of the Microsoft Atlas ti software (version 8) program.

**Results:**

The study results reveal a range of circumstances under which drowning occurs in Uganda, poor record keeping of drowning incidents, fear of reporting drowning incidences to the authorities, challenges in preventing drowning and proposed strategies for mitigating the problem.

**Conclusions:**

This study found that there is no specialized record keeping system for drowning cases in Uganda and where such records are kept, the system is entirely manual (in hard copy form) with no electronic storage of data. Secondly, the drowning cases reported to police posts and stations in various parts of the country are not transmitted to the district headquarters and national database. These and other conclusions not only provide valuable insights into understanding of drowning circumstances but also the key policy and programme interventions for water-based economic activities such as fishing and public water transportation in Uganda and other LMICs.

## Background

Although drowning ranks highly among the leading causes of morbidity and mortality globally, it suffers from underreporting and limited public health advocacy [1]. In proportion to the total drowning deaths worldwide, unintentional drowning-associated mortality was generally higher in children, males and in low-Social Demographic Index (SDI) to middle-SDI countries for example, China, India, Pakistan and Bangladesh accounted for 51.2% of all drowning deaths in 2017. Oceania was the region with the highest rate of age-standardised YLLs in 2017, with 45 434 (40 850 to 50 539) YLLs per 100 000 across both sexes [2]. Despite increasing focus by researchers in understanding the epidemiology of drowning injuries, there is a paucity of empirical evidence on the underlying locally based primary and secondary circumstances under which it occurs. Data on drowning deaths in LMICs are potentially underrepresented as a result of limited or inaccurate data collection and insufficient continuity of data [3, 4]. Some of the underlying reasons for underrepresentation include the exclusion of intentional drowning by suicide and homicide, as well as drowning cases related to natural disasters and water transport incidents [5].

Although a combination of infrastructure, geographical barriers and socio-economic factors has been cited in the failure to report drowning cases and keep updated records of such incidents in most LMICs [6], the extent to which these and other intervening variables affect the drowning record keeping processes remains a matter of contestation. In Africa, it is likely that traditional beliefs, customs and values in some countries play an important role in the decision-making process—so much so that African kin groups are sometimes described as communities of both the living and the dead [7]. It is a complex scenario—as researchers explore the reporting of drowning cases to different sources such as superstition and witchcraft and the linkage of cases across these sources in order to make recommendations for drowning reporting and surveillance.

In addition to the limited literature on the extent and trends of intentional drowning deaths even in high-income countries where injury surveillance is better established than in developing nations [8], most studies on intentional drowning explore suicides by drowning, with limited attention on submersion deaths due to assault [9]. Similarly, research in most LMICs rarely focuses on the role of chronic diseases and risk conditions, such as cardiac rhythm disorders, hypertension, diabetes mellitus, anxiety, autism or acute conditions like myocardial infarction, or epileptic seizures in drowning [10]. So, whereas the etiology of drowning is widely considered to be multifactorial and varies with age and geographic location [11], the overall incidence of such cases, reporting rates and record keeping systems differ from one country to another.

In Africa, the full burden of drowning is likely underestimated as routine surveillance data are lacking, resulting in a poor knowledge base for the development of prevention strategies [12]. However, the Lake Victoria region and the countries that surround it—Uganda, Kenya, Tanzania—have very high rates of drowning amongst the lakeside communities, but also along rivers, and substantially fewer drownings in areas where the residents are not exposed to large natural water bodies [13]. According to the country health profile for Uganda published by the World Health Rankings, drowning claimed a total of 3,186 lives in 2017, representing 1.23 percent of the total deaths that year. However, there is limited information on the burden of drowning due to the lack of a systematic process to record and track drowning-related incidents and deaths in Uganda, [14]. Similarly, activities of boat transportation and commercial fishing have contributed to drowning and there are neither community studies nor mandatory death registrations to drowning-related data [15]. The appropriateness and applicability of these interventions in Uganda is unknown. To reduce drowning in Uganda, it is essential to implement proven strategies tailored to the unique circumstances of drowning in the country.

The aim of this paper was to draw on the results of semi-structured interviews with witnesses, family members and friends of persons involved in fatal and nonfatal drowning incidents to describe the circumstances of drowning in both lakeside and non-lakeside districts and to identify potential contextually appropriate interventions for drowning prevention and surveillance in Uganda.

### Study area and methods

The study was conducted in the following 14 districts in Uganda: Masaka, Hoima, Mayuge, Arua, Kampala, Kitgum, Mbarara, Mubende, Serere, Lira, Rubirizi, Nakasongola, Soroti and Rakai. The combined population in these districts is estimated to be approximately 7,187,276 people, which is about 19% of the total population of Uganda [16]. This was part of a two-phased study which was based on separate quantitative (phase one) and qualitative (phase two) approaches. For this particular phase, the 14 study districts were selected through purposive sampling from the 60 districts where the study phase one data collection occurred to ensure that both lakeside and non-lakeside districts were included. The sampling also ensured that each geographical region (Central, Eastern, Northern, and Western) was represented. In addition, the districts were selected to include those with high and low drowning burden as identified during phase one of the study. For feasibility, the number of languages spoken in the district and accessibility of the district were taken into consideration.

The qualitative research team was led by a qualified and experienced expert in qualitative research data collection and analysis and comprised 12 selected research assistants. The data collection process started by contacting the 14 Health Assistants in the selected districts to identify all cases of drowning that occurred during a two-and-a-half-year period (between January 1, 2016 and June 30, 2018). Sixteen interviews were held with the data stewards in district police offices in the study districts. In addition, a total of 324 face-to-face interviews were held with witnesses/family members/friends of and survivors of drowning incidents to determine the circumstances of drowning. Finally, 10 focus group discussions (FGDs) were held with community members to explore cultural factors and beliefs associated with drowning and potential interventions as follows: Mayuge (2), Serere, Kitgum, Rakai (2), Mbarara, Arua, Masaka and Kampala (2). The FGDs comprised 8–10 members who were equally balanced between male and female study participants. The FGD guides were developed in English and translated and back-translated into all the local languages spoken by communities in the study areas by certified language translation experts. Each FGD and individual interview lasted an average of an hour and a half. Both the key informant and FGD guides were pre-tested on communities that had not been included in the study sample population to eliminate ambiguity and other issues before they were used on the target population. Similarly, all the interview recordings were done in the local language before being translated into English by the research assistants ahead of the analysis process. The research team also held a data validation and verification session in each of the study districts [17]. Such sessions involved engaging the study respondents in discussing the study results to ensure that they reflected their views, were valid and ably answered the research questions posed to them during the interviews.

**Fig. 1:**
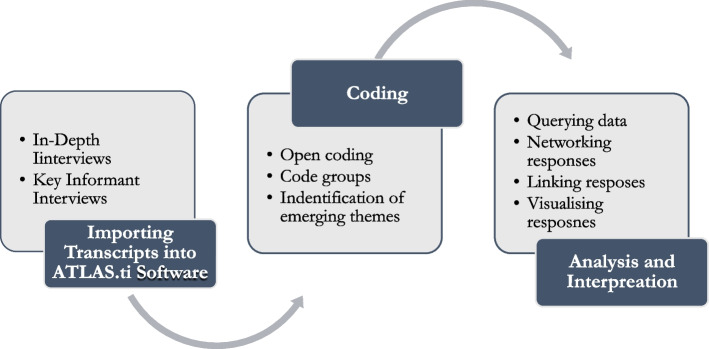
Data analysis process

Data analysis was done at two levels: First, data from the FGDs and the data steward interviews were conducted using the Framework Analysis approach [18]. Among the key tenets of the Framework Analysis Approach (FAA) were acquainting ourselves with the data (often referred to as familiarization), identifying the themes and interpretation of the data. After transcribing both sets of interviews, the transcripts were read and re-read before constructing themes based on the specific objectives of this phase of the study. Secondly, the in-depth interviews with individual family members and/or friends and relatives who survived or witnessed the drowning incidents were analysed using the Microsoft Atlas ti software (version 8) program. The software was chosen because of its ability in facilitating analysis of qualitative data and its capability to import, sort and facilitate analysis of rich text and plain text documents. For all the qualitative data collected for the study, three major themes developed namely: causes of drowning, circumstances of drowning and prevention of drowning. The steps followed are illustrated in Fig. 1.

All primary data were imported into ATLAS.ti software while secondary data in form of published articles in peer reviewed journals were also imported into the software for further analysis. By and large, the framework enabled the creation of a new structure for the data (rather than the full original accounts given by the study participants) that we found quite helpful in summarizing/reducing the data in a way that supported answering the research questions. Besides, codes were—during the analysis process–grouped into clusters around similar and interrelated ideas or concepts while categories and codes were arranged in a tree diagram structure in the analytical framework.

The secondary data were obtained in the form of published peer-reviewed journal articles and official documents obtained from databases searched between July 2018 and December 2019: Wiley Online Library, Google Scholar and Ebscohost. A total of 82 articles that met the inclusion criteria after reviewing the abstract or full text were included based on: being written in English, published between 2014 and 2018 and included a measure of, or report on drowning, water transport and drowning survivor. The analysis was preceded by coding of all documents imported into the software. Coding is a way of indexing or categorizing text in order to establish a framework of thematic ideas about it [19]. Most importantly, this study focused on axial coding that involved relating data together in order to reveal codes, categories, subcategories and emerging themes. However, the study also used pre-determined codes (code book) which included: Station Diary and Records Office (reporting drowning), fishing methods and weather conditions (drowning circumstances) and water transport policy and community sensitization (drowning prevention interventions). Inductive and deductive approaches of coding reinforced the data coding process [20].

During the data analysis process, the study team selected and included specific quotes in the results that were illustrative of the identified themes.

### Study findings

The study findings fall into the following themes: reporting and recording drowning incidences, describing drowning circumstances and identifying potential interventions for the prevention and surveillance of drowning.

### Reporting and recording drowning incidents

The record keeping system for drowning cases was arguably one of the starting points in understanding the underlying circumstances under which drowning incidents occur and a key reference to identifying potential contextually appropriate interventions. In Uganda, the system starts with entering the drowning incident reported in a Station Diary found at all police posts and stations throughout the country. The initial record is entered by any police officer on duty at the station. In cases where the incident report bears the hallmarks of criminality, a Desk Inquiry File (DIF)—also referred to simply as Death File by some data stewards—is opened and forwarded to the counter (reception). The file is then sent to the Records Office for registration and delivered to the Officer in Charge, Criminal Investigations Department (CID) who allocates it to a homicide squad officer to proceed with the investigations. Later, the file is sent to the Resident State Attorney (RSA) for perusal and to determine whether or not there is sufficient evidence to constitute criminal proceedings against any of the parties involved in the drowning incident.

Overall, there was no specific book in which drowning cases are recorded. Such reports are often entered by hand into the Station Diaries just like any other crimes reported at a particular police post or station. Only a few of them that merit further investigations are isolated at some stage—a view highlighted by a data steward from the central region who preferred anonymity.

*“We have the police Form 1 where we record all kinds of death such as death by drowning, death by shooting and all that. We file all sorts of deaths…including accidents without separating them.”*

Whereas the majority of the data stewards indicated that all the drowning cases recorded at the local police stations were sent to the offices of the District Police Commanders (DPCs) or the Officers-in charge, Criminal Investigations Department (CID), some of them indicated that not all cases follow the legally established system. They cited a breakdown in communication and negligence on the part of some police personnel at the local stations who might either be bribed not to forward their files or simply fail to forward the case to the district police station. In Kampala, the case management office for handling drowning investigations is found at the Divisional headquarters which is the equivalent of a political constituency (county) in other parts of the country.

Regarding the use of drowning data, police and other institutions of government use drowning data for a variety of purposes. Data stewards discussed various uses of data on drowning cases such as a basis for planning for prevention strategies. Based on the frequency of drowning cases reported at a particular police post or station, police and other government departments are in position to map out the trends of drowning incidents and the key ‘hotspots’ on various water bodies and devise appropriate interventions to reduce such incidences.

*“Data on drowning really gives us a yardstick on where to concentrate our efforts to prevent more of such (drowning tragedies),”* –Data Steward in Eastern Uganda.

In addition, such data also feed into the national crime (monthly, quarterly, bi-annual or annual) reports which are presented to the media and submitted to parliament by the Inspector General Police (IGP) and for reference where court proceedings are instituted regarding a particular drowning incident (such as allegations of foul play in the drowning incident). Police also rely on such information in designing community policing sensitization campaigns such as the need to wear lifejackets while using water transport on all major water bodies. There was also evidence of the use of drowning data to inform public health research projects by institutions such as the Makerere University School of Public Health and Centre for Disease Control and Prevention (CDCP).

As far as reporting drowning cases is concerned, the Uganda Public Health Act (as amended) compels every individual to first report every unnatural death to police before the burial takes place. In such circumstances, a postmortem examination is always undertaken and a death certificate issued. Study participants discussed a range of context-specific incentives and hindrances to reporting drowning cases in Uganda. Some of the motivating factors for reporting drowning incidences to police are the expectation for free transport of the drowning victim’s body by police from drowning scenes to burial sites and potential free embalmment drowning victims’ bodies by the police surgeon and mortuary pathologists. On the other hand, the study participants referred to a common belief among many Ugandans that drowning is—like many other public health issues—a God-sent phenomenon that does not warrant filing a police report.

*“Some of our people believe that ‘why should we bother going into all this bureaucracy by informing the police. Let us retrieve our body and we go and bury. After all, we all know our person has died naturally according to God’s will. There is no any other outside force that has caused the death,”*– Data Steward in Eastern Uganda.

In fishing communities, there is also a widespread belief that an individual who dies in a drowning incident on a water body should not be reported to police because he died in the line of duty. Furthermore, individuals who come across floating unidentified bodies on water bodies might also fear to report for lack of detailed information on the victim(s) and the drowning circumstances. Other obstacles to reporting drowning incidences cited were the following: the fear of being asked to pay for a postmortem examination fee, lack of trust in the police’s investigation system and the physical infrastructure and geographical barriers which make it difficult for people living in hilly and mountainous areas to travel to the nearest police stations to report such cases.

Even in circumstances where most drowning incidences are reported to police, this study also highlighted a host of challenges that undermine the record keeping system. These include lack of or limited office space for data stewards, limited funding to the office of data stewards (resulting into failure to buy vital supplies and equipment such as stationery, gloves, cameras, hydraulic cutters, walkie talkies for communication, protective clothing and other materials for the police investigations department) and computers to digitize the hard copy files that pile up in the police records steward offices every other year. The record keeping system also undermines confidentiality in handling sensitive data on drowning cases as it is recorded in the same book (the Station Diary) together with all crimes.

### Describing drowning circumstances in Uganda

The study participants discussed a range of circumstances under which drowning occurs in Uganda. They pointed to situations where some communities use fishing methods that render them highly vulnerable to drowning. For example, in the Busoga region, interviewees in a focus group discussion noted that the use of fishing nets locally known as ‘ponyoka’ (just throw) while wearing a shirt with buttons was a recipe for a drowning incident. *“When an individual wearing a shirt with buttons throws the net to trap the fish, the fishing net gets stuck in the button and it drags the individual into the water. Within no time, such a person would be dead,”* explained an FGD participant held at Bwondha landing site on Lake Victoria.

Drowning also occurs during the unpredictable heavy storms caused by strong winds that often sweep away boats and canoes on the lakes and rivers. Such winds are often responsible for easily overturning weak and small ‘three pieces of timber’ boats (locally known as ‘parachutes’ or ‘para’ or ‘bawo tatu’). Drowning incidents also take place in situations where the fishermen use illegal fishing methods and illegal gear, and they and attempt to flee from arrest by security personnel. Similarly, drowning often occurs when fishing or water travel takes place in areas that are used as habitats for potentially dangerous water animals such as crocodiles, rhinoceroses and large snakes (notably the puff adders and pythons).

This study also brought up unique circumstances of drowning that some study participants attributed to ‘destiny,’ ‘bad luck,’ ‘the devil *(Lucifer)* in the water’ and suicidal tendencies. This was especially highlighted among women who face domestic violence, marital issues, unwanted pregnancies, mental illness, living with a terminal illness or stress from failure to service loans and mortgages—as explained by one 60-year-old male participant during an FGD.

*“There are many people who are suicidal. When they get a problem with their spouses or neighbor, they can decide to walk into the river or lake and die after falling in the water.”*

Other circumstances such as the nodding disease syndrome in parts of Northern Uganda (locally known as *lucluc*) were also cited as resulting in drowning when affected children fall into ponds, wells and major water bodies, and the non-use of quality lifejackets during fishing or water transport activities.

### Identifying potential interventions for drowning prevention and surveillance

Overall, the study participants highlighted a lack of policy and supporting infrastructure in their districts for drowning prevention and surveillance. For instance, where children aged under 18 are not allowed to swim or go for fishing expeditions in the waters, there is not supporting legislation to enforce this. There is also no supporting infrastructure to train or rescue individuals who travel or their conduct economic activities in the lakes and rivers as explained by one 32-year-old male participant during an FGD.

*“Our government just talks but there is no law and action. Even when they arrest someone for endangering his or her life in the water, they can’t be taken to court. There is no specific law. There are also no guidelines for swimming around this area. And then, the worst bit is that there are no arrangements to provide life jackets or even train boat riders and other water passengers on prevention of drowning.”*

The lack of political will and competing demands for resources were largely cited as some of the key factors hindering the promulgation of an enabling policy framework—a view amplified by one female politician during an interview:

*“Quite simply, our government’s interests lie in funding politicians and other people who support their stay in power. That is where the resources are allocated; not in preventing drowning. They don’t care that much.”*

## Discussion and policy implications

One of the key objectives for this study was to examine the record keeping system for drowning incidences in Uganda. Findings show that whereas most people are fully aware of the mandatory need for reporting, some drowning cases go unreported especially in cases where the victim’s body can be easily retrieved and hastily buried before police are notified. There was also evidence that some people only inform police after retrieving a drowning victim’s body. This study also demonstrated that some people prefer not to subject the bodies of drowning victims largely because such processes are lengthy and involve hidden costs which they cannot afford.

The study findings also highlighted several incidences of children drowning in non-lakeside districts. Although there is a paucity of literature describing drowning epidemiology in Uganda, data provided by interviewees in this study showed that children account for an unspecified proportion of fatal drowning incidents that occur in large, open bodies of water. They were also found to be highly vulnerable in places where there are drainage channels such as Kampala and Mbarara (River Rwizi). Other drowning cases involving children included those in domestic water tanks, buckets and basins. Whereas this study was not necessarily about drowning by children, the findings point to the need to focus on child-specific policy and programme interventions such as the inclusion of swimming lessons and heightened supervision of swimming pools and other water bodies to minimize cases of children drowning in Uganda. These findings are consistent with those of a related survey-based study in South Africa which showed that 52.2% of fatal drowning incidents occurred on weekdays and 47.8% occurred on weekends and public holidays [21]. The same study further provided a set of developmental characteristics and multiple contextual factors which put children living in low-income countries such as Uganda at increased risk for drowning. Similarly, studies in Ethiopia, Guinea, Ivory Coast, and Nigeria have provided empirical evidence that older children and young adults may be more at risk of drowning, as opposed to toddlers [22–25]. Additional studies in Sub-Saharan Africa such as one in Tanzania have corroborated these findings by suggesting that the rates of drowning death among young children vary based on location (rural versus urban) and by sex within the same country [26]. Other studies that have examined locations and risk factors for drowning have also pointed to a need for good epidemiological studies across all African countries to describe the patterns of drowning and understanding of the risk factors and an evaluation of the prevention strategies [27, 28].

Even if it is often difficult to draw a clear distinction between the causes and circumstances of drowning, the drowning circumstances that emerged from this study re-ignited the discussion on intentional and unintentional drowning. Much as this paper does not provide any statistical evidence on the mortality and morbidity rates of suicide drowning in Uganda, study participants narrated various incidences in which the drowning victims either died or sustained injuries by drowning. Therefore, while suicide rates may not be as significant as those of the widely known traditional forms of suicide such as poisoning and suffocation, they provide a key pointer for relevant policy and programme design and implementation for drowning prevention. This is akin to findings in Australia where unintentional drowning deaths were found to only be part of the drowning profile, with little attention being paid to intentional drowning and the strategies for the prevention of intentional drowning deaths likely to be different from unintentional [29].

The findings further show that most respondents feel that the government has failed to prioritise drowning among the major public health issues in the country. Drawing parallels to its response to the Ebola outbreaks, the government is seen as often acting indifferent to addressing the major causes of drowning in Uganda. In addition to its failure to enforce the existing water transport policies, government is also widely blamed for refusing to provide high quality lifejackets and adequate marine control units on the major water bodies. Government is also blamed for failing to stop the inhumane treatment by the Uganda Peoples’ Defence Forces (UPDF) of fishermen on the water bodies as they try to curb illegal fishing.

Through the Ministry of Works and Transport, and that of Water and Environment, the central government is responsible for oversight and overall policy guidance to the water transport sector. It provides a basis for establishing a safe water transport policy environment on all water bodies. Key among the required policy interventions is the need to increase the budget for the provision of safe water sources from 3.3% of the total national budget in the 2019/20 Financial Year to a higher percentage in line with internationally recommended levels of funding.

At the sub-national level, this study has shown that most districts are greatly resource-constrained and, therefore, have limited capacity to enforce measures to reduce drowning incidences. Faced with many competing needs—largely administrative and emergency (disaster related)—most local governments often have no financial resources to allocate towards the prevention or mitigation of drowning in their localities. Although they can independently enact and enforce bylaws and ordinances to prevent drowning, there is limited capacity for the councilors to embark on this process. In terms of enforcement, they were still found to be limited to only two landing sites. Although the enactment and enforcement of bylaws and ordinances was widely recommended as critical for the prevention of drowning, this requires a high level of commitment from district leaders and mobilization of funds to operationalize the strategy. Furthermore, the roles of the different actors in operationalizing this strategy should be well articulated and explained. There is also limited political will to focus on drowning in most districts—especially in the non-lakeside areas where drowning incidences are seldom reported. One of the major problems for most districts is to identify and pursue the most effective and efficient strategy for preventing drowning with their limited finances. This therefore calls for strategic planning based on empirical evidence in setting targets and enforcing measures to improve water transport safety.

On its part, the community leadership at the village, parish and sub-county levels needs to heighten sensitization on the risks and prevention of drowning. Specifically, leaders must champion the sensitization of local residents on avoiding leaving ditches, holes and other open spaces where water can collect uncovered. Community sensitization should occur in tandem with the enforcement of bylaws prohibiting the use of drugs and consumption of alcohol by fishermen and water transport users prior to and during water-related activities and the demystification of the traditional beliefs, practices and myths that often shroud some of the drowning circumstances highlighted in this paper. Such strategies have also been successful in preventing other public health issues in low-and middle-income countries such as Nepal [30].

### Study limitations

The purposive sampling technique used to identify witnesses/family members/friends of drowning cases could have the potential for sampling biases and limited control over the sampling method by the researcher that may have an effect on the study findings. The authors of this paper are also fully aware that the findings of this study can be transferred to another setting, context or group even if they are based on qualitative interviews. Even if they cannot be used to compare the drowning burden in the study areas with other places however, they provide key information about much needed efforts to prevent drowning in Uganda. Despite these limitations however, this qualitative study paper will make a significant contribution to the dearth of literature in this important field of public health research.

## Conclusions

One of the key conclusions from this qualitative component of the study was that there is no specialized record keeping system for drowning cases in Uganda. The system is entirely manual (in hard copy form) with no electronic storage of data reported anywhere in the districts where this study was done. Secondly, the study demonstrated some of the drowning cases reported to police posts and stations in various parts of the country are not transmitted to the district headquarters which explains the disparity between the numbers of drowning cases found by the research teams at the districts or major mortuaries in Phase one and those found either at the local police posts or in the villages identified during Phase two of the study. Finally, the study provided insights into drowning circumstances that can inform policy and programming for fishing and public water transportation in Uganda and other LMICs. Given that many interviewees in this study feel that the Government of Uganda does not consider drowning as one of the major public health issues such as Ebola (or even COVID-19), the evidence provided herein can greatly contribute to challenging the limited consideration for the effects of the drowning burden in Uganda and other developing countries.

One of the key take home messages from this study is a need to support surveillance and the prevention of drowning in all the major water bodies in the country. Policy makers should also provide more budgetary support for the provision of safe water sources, strengthened strategic planning and the use of community leadership to sensitise the population on the risks of drowning.

## Data Availability

The datasets generated and/or analysed during the study are not publicly available because they are huge files which are safely locked away from any individual except on special request from the corresponding author under special circumstances.
